# Supplementary material for: Dissipation-driven selection of states in non-equilibrium chemical networks
Source: Commun Chem. 2021 Feb 15;4:16. doi: 10.1038/s42004-021-00454-w (PMC9814615; doi:10.1038/s42004-021-00454-w)
Supplement: Supplementary file 2 — Supplementary Information [file 42004_2021_454_MOESM2_ESM.pdf]

Supplementary Information for "Dissipation-driven selection of states in non-equilibrium chemical networks"

Daniel M. Busiello,[1] Shiling Liang,[1] Francesco Piazza,[2,3] and Paolo De Los Rios[1,4]

[1]*Institute of Physics, Ecole Polytechnique Fédérale de Lausanne (EPFL), 1015 Lausanne, Switzerland*
[2]*Centre de Biophysique Moléculaire (CBM), CNRS-UPR 4301, Rue C. Sadron, Orléans 45071, France*
[3]*Université d'Orléans, Chéteau de la Source, Orléans Cedex 45071, France*
[4]*Institute of Bioengineering, Ecole Polytechnique Fédérale de Lausanne (EPFL), 1015 Lausanne, Switzerland*
(Dated: January 8, 2021)

I. SUPPLEMENTARY METHODS

Details about Three-states two-box model

In the main text, we characterize the model by defining the relations among transition rates (Eqs. (2) and (3) of the main text). However, when the system is driven away from equilibrium by a temperature gradient, it is necessary to set a value for each rate to solve the dynamics and find the stationary state. Throughout the article, we use:

$$\begin{aligned} k_{A_i \to B_i} &= e^{-\Delta E/k_B T_i} \\ k_{A_i \to C_i} &= e^{-\Delta E/k_B T_i} \\ k_{B_i \to A_i} &= e^{-\Delta E - \Delta\epsilon_B/k_B T_i} \\ k_{C_i \to A_i} &= e^{-\Delta E - \Delta\epsilon_C/k_B T_i} \end{aligned} \tag{S1}$$

with $i = 1, 2$. Remarkably, this choice is not unique, and all other choices do not change the whole message of the work. A hint about the robustness of the message is that, for small gradient, only the difference between the barrier $\Delta\epsilon$ appears (Eq. (5) of the main text). The choice in Eq. (S1) has the advantage that all rates stays finite in the limiting case of infinite barriers, resulting in a finite entropy production.

Three chemically interacting species with diffusion

Here, we present some mathematical details about the continuous version of the discrete-state model. The dynamics, presented in the main text, follows a differential Chapman-Kolmogorov equation:

$$\partial_t p_X(x) = \sum_Y (k_{Y\to X}(x) p_Y(x) - k_{Y\to X}(x) p_X(x)) + D_X \partial_x^2 p_X(x) \tag{S2}$$

where $X, Y = A, B, C$ and $x \in [0, 1]$ with boundary conditions $\partial_x p_X(0) = \partial_x p_X(1) = 0$, i.e. no flux at the boundary. For sake of simplicity, we set $D_A = D_B = D_C = D$, as for the discrete-state outlined in the main text. Let us recall that the rates governing the transitions are:

$$\begin{aligned} k_{A\to B}(x) &= e^{(E_A - E_B)/k_B T(x)} k_{B\to A}(x) \\ k_{A\to C}(x) &= e^{(E_A - E_C)/k_B T(x)} k_{C\to A}(x) \end{aligned} \tag{S3}$$

with the additional condition on the energy barriers:

$$k_{C\to A}(x) = e^{\Delta\epsilon/k_B T(x)} k_{B\to A}(x) \tag{S4}$$

where the barrier $\Delta\epsilon = \Delta\epsilon_B - \Delta\epsilon_C > 0$, so that the transition involving A and C is faster than the one involving A and B. Here, the probability to find the system in a given state X, independently of the position, is:

$$P(X) = \int dx p_X(x) \qquad X = A, B, C \tag{S5}$$

We will use small $p_X(x)$ for space-dependent probabilites, and capital $P(X)$ for their space integrated counterpart.

We have already seen in the main text that, in equilibrium conditions, the system asymptotically converges to $P_B^{eq} = P_C^{eq} > P_A^{eq}$. If there is no temperature gradient, the system relaxes to the Boltzmann distribution determined by its global temperature T for all the species. On the contrary, in case of no diffusion, the probability for each species is Boltzmann distributed as a function of the local temperature $T(x)$.

Limit $D \to \infty$

Following the standard approach of the time-scale separation [1, 2], it is possible to define a set of effective transition rates, $\hat{k}_{X\to Y}$, such that the stationary solution, in the limit $D \to \infty$, is given by the one of a discrete-state system subject to these $\hat{k}_{X\to Y}$, without diffusion. Naively speaking, dividing the space in n boxes, we are imaging that for $D \gg k_{X\to Y}$ each species will feel the contribution from each box at the same time, performing then transitions at an effective rate given by:

$$\hat{k}_{X\to Y} = \int dx \mu(x) k_{X\to Y}(x) \tag{S6}$$

where $\mu(x)$ is the probability distribution satisfying $D_X \partial_x^2 P(X(x)) = 0$, which is uniform in x. Then, the solution for each species can be readily found:

$$\begin{aligned}
P(A) = \int dx\; p_A(x) &= \frac{\hat{k}_{B\to A}\hat{k}_{C\to A}}{\hat{k}_{B\to A}\hat{k}_{A\to C} + \hat{k}_{C\to A}\hat{k}_{A\to B} + \hat{k}_{C\to A}\hat{k}_{B\to A}} \\
P(B) = \int dx\; p_B(x) &= \frac{\hat{k}_{C\to A}\hat{k}_{A\to B}}{\hat{k}_{B\to A}\hat{k}_{A\to C} + \hat{k}_{C\to A}\hat{k}_{A\to B} + \hat{k}_{C\to A}\hat{k}_{B\to A}} \\
P(C) = \int dx\; p_C(x) &= \frac{\hat{k}_{B\to A}\hat{k}_{A\to C}}{\hat{k}_{B\to A}\hat{k}_{A\to C} + \hat{k}_{C\to A}\hat{k}_{A\to B} + \hat{k}_{C\to A}\hat{k}_{B\to A}}
\end{aligned} \tag{S7}$$

This solution gives exactly the same result as in Eq. (5) of the main text.

Perturbation theory approach

It is interesting to explore in details the limit of small gradients for the continuous case. The perturbative approach that we are going to mention is valid for any form the transition rates. Then, in order to keep the analysis as general as possible, we simply say that:

$$\kappa(x) = \frac{k_{A\to B}(x)}{k_{B\to A}(x)} = \frac{k_{A\to C}(x)}{k_{C\to A}(x)} > 1 \qquad\qquad \alpha(x) = \frac{k_{A\to C}(x)}{k_{A\to B}(x)} > 1 \tag{S8}$$

Note that here α plays the same role as the energy barrier $\Delta\epsilon$. Eventually, we restrict ourselves to the physical choice of the Arrenhius' form when comparing these results with the one for the discrete case.

The temperature dependence appears only in the transition rates. Then, assuming that the temperature gradient is constant, *i.e.* $T(x) = T_0 + \Delta T \cdot x$, we can expand each of them as follows:

$$k_{X\to Y} = \sum_n \frac{1}{n!} x^n \nabla T^n \partial_T^n k_{X\to Y}|_{\nabla T = 0} \tag{S9}$$

From now on we will not write explicitly the fact that the expansion coefficient are evaluated at $T(x) = T_0$.

We can perform the same expansion also on the probabilities, as $p_X(x) = \sum_n \nabla T^n p_X^{(n)}(x)$. Substituting this into the dynamical equation, and using the fact that $p_A(x) + p_B(x) + p_C(x)$ is uniform at stationarity (since $D_A = D_B = D_C = D$), and equal to 1 for simplicity in a $1D$ box of unitary length, we get that the following n-th order set of equations for the steady state has to be fulfilled:

$$D\partial_x^2 p_X(x) = \sum_{m+l=n} \frac{1}{m!} x^m \left(\left(\partial_T^m k_{A\to X} + \partial_T^m k_{X\to A}\right) p_B^{(l)}(x) + \partial_T^m k_{A\to X} p_C^{(l)}(x) \right) - \frac{1}{n!} x^n \partial_T^n k_{A\to X} \tag{S10}$$

for $X = B, C$ only, with the boundary conditions $\partial_x p_X^{(n)}(x) = 0$ at $x = 0, 1$. For sake of simplicity, from now on we use the following positions:

$$\mathcal{K}_n^{(X)} \equiv \partial_T^n k_{A\to X} \qquad\qquad k_n^{(X)} = \partial_T^n k_{X\to A} \tag{S11}$$

Then we can solve Eq. (S10) in terms of the probability coefficients $p_X^{(n)}(x)$. It is worth showing the zeroth and first order corrections separately, since they elucidates some features of the system.

Zeroth order solution

At the zeroth order, as reported also in the main text, we obtain:

$$p_B^{(0)}(x) = p_C^{(0)}(x) = \frac{\kappa(x)}{2\kappa(x)+1} \tag{S12}$$

Then $P^{(0)}(B) - P^{(0)}(C) = 0$ at the lowest order, meaning that, as expected, a thermal gradient is needed to allow reaching a non-equilibrium stationary state. Here $P^{(n)}(X)$ is the integral of $p_X^{(n)}(x)$ over the whole domain.

First order solution

In order to get the first order solution, we have to solve Eq. (S10) with $n = 1$. For sake of clarity, let us introduce the following constants:

$$U_n^{(X)} = \frac{K_n^{(X)} + k_n^{(X)}}{D} \qquad\qquad V_n^{(X)} = \frac{K_n^{(X)}}{D} \tag{S13}$$

Rewriting the dynamical equation in terms of these quantities and $P_0(X(x))$, by direct integration, we get:

$$p_X^{(1)}(x) = 2\left(M_X^+ - M_X^-\right)x + 2M_X^+ L^+ \frac{\sinh\left(\frac{1-2x}{2L^+}\right)}{\cosh\left(\frac{1}{2L^+}\right)} - 2M_X^- L^- \frac{\sinh\left(\frac{1-2x}{2L^-}\right)}{\cosh\left(\frac{1}{2L^-}\right)} \tag{S14}$$

where, with X always intented as B, C:

$$\begin{aligned}
W^{(X)} &= U_1^{(X)} P_0(B(x)) + V_1^{(X)} P_0(C(x)) - V_1^{(X)} \\
J &= \sqrt{\left(U_0^{(C)} - U_0^{(B)}\right)^2 + 4V_0^{(B)}V_0^{(C)}} \\
L^\pm &= \sqrt{\frac{2}{U_0^{(C)} + U_0^{(B)} \pm J}} \\
M_B^\pm &= \mp\frac{(L^\pm)^2}{2}\left(W^{(B)}\frac{\mp U_0^{(C)} \pm U_0^{(B)} + J}{2J} \pm W^{(C)}\frac{V_0^{(B)}}{J}\right) \\
M_C^\pm &= \mp\frac{(L^\pm)^2}{2}\left(W^{(C)}\frac{\mp U_0^{(C)} \pm U_0^{(B)} + J}{2J} \pm W^{(B)}\frac{V_0^{(C)}}{J}\right)
\end{aligned} \tag{S15}$$

Considering the physical situation in which we have the Arrhenius's form for the transition rates [3–5]:

$$p_C^{(1)}(x) - p_B^{(1)}(x) = \left(M_B^+ - M_C^+\right)\left(2L^- \frac{\sinh\left(\frac{1-2x}{2L^-}\right)}{\cosh\left(\frac{1}{2L^-}\right)} - 2L^+ \frac{\sinh\left(\frac{1-2x}{2L^+}\right)}{\cosh\left(\frac{1}{2L^+}\right)}\right) \tag{S16}$$

Most importantly, this expression is symmetric around $x = 1/2$ in our domain of length 1. This means that the unbalance between state C and B is independent of the sign of the gradient.

Second order solution for $\Delta\epsilon \to 0$

The n-th order solution can be readily found by direct integration of the n-th order dynamical equations, getting:

$$P^{(n)}(C) - P^{(n)}(B) = \frac{1}{U_0^{(B)}U_0^{(C)} - V_0^{(B)}V_0^{(C)}}\left((U_0^{(B)} + V_0^{(B)})\int_0^1 dx\; g_n(x) - (U_0^{(C)} + V_0^{(C)})\int_0^1 dx\; f_n(x)\right) \tag{S17}$$

It is clear that this expression is well defined once one knows the solution at the lower orders.

The second order solution can be obtained by specializing Eq. (S17) with $n = 2$ and specifying the functional form of $f_2(x)$ and $g_2(x)$ as:

$$f_2(x) = \frac{x^2}{2}\left(U_2^{(B)}p_B^{(0)}(x) + V_2^{(B)}p_C^{(0)}(x) - V_2^{(B)}\right) + x\left(U_1^{(B)}p_B^{(1)}(x) + V_1^{(B)}p_C^{(1)}(x)\right)$$
$$g_2(x) = \frac{x^2}{2}\left(U_2^{(C)}p_B^{(0)}(x) + V_2^{(C)}p_C^{(0)}(x) - V_2^{(C)}\right) + x\left(U_1^{(C)}p_B^{(1)}(x) + V_1^{(C)}p_C^{(1)}(x)\right) \tag{S18}$$

We note that all the terms that do not scale with the diffusion have to cancel out. Indeed, when $D \to 0$, there is no difference between $P(C)$ and $P(B)$, as they have the same energy and the system relax to the Boltzmann distribution. Then, after some calculations, taking, for sake of simplicity, $\Delta E \equiv E_A - E_B$ and $E_B = E_C$, we obtain:

$$\begin{aligned} P^{(2)}(C) - P^{(2)}(B) \; = \; & \frac{\Delta E}{2T_0^4}\frac{V_0^{(B)}}{U_0^{(B)} + V_0^{(B)}}\left(\frac{U_0^{(C)} - U_0^{(B)} - 2V_0^{(C)}}{J} + 1\right)\Delta\epsilon\;(L^-)^2\left(1 - 2L^-\tanh\left(\frac{1}{2L^-}\right)\right) + \\ & - \frac{\Delta E}{2T_0^4}\frac{V_0^{(B)}}{U_0^{(B)} + V_0^{(B)}}\left(\frac{U_0^{(C)} - U_0^{(B)} - 2V_0^{(C)}}{J} - 1\right)\Delta\epsilon\;(L^+)^2\left(1 - 2L^+\tanh\left(\frac{1}{2L^+}\right)\right) \end{aligned} \tag{S19}$$

In order to interpret this formula, we consider the limit $\Delta\epsilon \to 0$, i.e. the energy barrier discriminating between fast and slow states is small. In this case,

$$K_0^{(C)} - K_0^{(B)} = K_0^{(B)}\frac{\Delta\epsilon}{k_\mathrm{B}T_0} + \mathcal{O}(\epsilon) \qquad\qquad k_0^{(C)} - k_0^{(B)} = k_0^{(B)}\frac{\Delta\epsilon}{k_\mathrm{B}T_0} + \mathcal{O}(\epsilon) \tag{S20}$$

Using this expansion we can compute the higher moments of the transition rates, and also the expressions for the quantities defined in Eq. (S15) up to the first order in $\Delta\epsilon$. Then, Eq. (S19), up to the first order in $\Delta\epsilon$, becomes:

$$P_C^{(2)} - P_B^{(2)} = \frac{\Delta E}{T_0^4}P_B^{eq}L_\mathrm{s}^2\left(1 - 2L_\mathrm{s}\tanh\left(\frac{1}{2L_\mathrm{s}}\right)\right)\Delta\epsilon > 0 \tag{S21}$$

with $L_\mathrm{s} = \sqrt{D/(k_{B\to A} + 2k_{A\to B})}$. As expected this difference vanishes when $D \to 0$, while, in the opposite limit $D \to \infty$, the characteristic length scale L_s tends to a constant value.

Scaling parameter $\sqrt{D/k_{A\to B}}$

Here we highlight the role played by the scaling parameter $\sqrt{D/k_{A\to B}}$, which is similar to what naturally arises as a characteristic length scale for the system, L_s, from the second order solution.

Although we have seen that the state C is globally favourable, for small values of the gradient ∇T, it is possible to find a region of parameter space in which:

$$P(C(x)) - P(B(x)) < 0 \tag{S22}$$

For sake of simplicity, let us assume that the forward reactions $k_{A\to B}$ is much faster than the reverse one $k_{B\to A}$, i.e. the energy difference ΔE is large. In this case, it is easy to see that, up to the first order in ∇T, we get:

$$p_C^{(1)}(x) - p_B^{(1)}(x) = (M_B^+ - M_C^+)\left(2L^-\frac{\sinh\left(\frac{1-2x}{2L^-}\right)}{\cosh\left(\frac{1}{2L^-}\right)}\right) \tag{S23}$$

This quantity decreases along the temperature gradient, meaning that it is at its lowest value and negative for $x = 1$. However, its integrated value still remains positive as we have shown above. In particular, the ratio between the second order and the first order contributions is:

$$\frac{p_C^{(2)}(x) - p_B^{(2)}(x)}{p_C^{(1)}(x) - p_B^{(1)}(x)} \propto \frac{1}{T_0}\sqrt{\frac{D}{k_{A\to B}}} \tag{S24}$$

involving the characteristic length scale L_s introduced above, when ΔE is large.

Numerical analysis beyond perturbation theory

In the main text we claimed the existence of an optimal gradient for selection both for the two-box model and for the continuous case. Here we corroborate this motivation with numerical simulations.

We find the stationary solution of the Master Equation (S2) numerically with no-flux boundary conditions, using the built-in solver of Mathematica. The "accuracy goal" has been set equal to half the Machine Precision (53 bits). We consider the presence of a linear temperature gradient, $D_A = D_B = D_C = D$, $\Delta E = E_A - E_B$, and $E_B = E_C$, which are the working conditions of the manuscript. In Fig. S1 we fix $T(0) = 0.2$, $\Delta E = 2$, and show that the ratio P_C/P_B as a function of ΔT exhibits an optimum for four different combinations of $\Delta\epsilon$ and D.

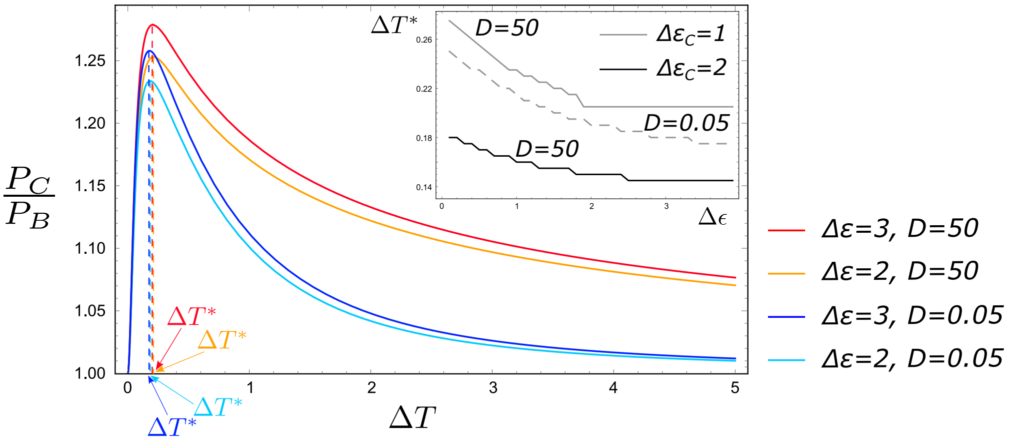

FIG. S1: Unbalance between P_C and P_B in the continuous model, quantified through the ratio $R_{CB} = P_C/P_B$, as a function of the gradient ΔT. We set $T(0) = 0.2$, $\Delta E = 2$ and two different values for energy barriers and diffusion coefficient D. In particular, $\Delta\epsilon = 3$, $D = 50$ (red curve), $\Delta\epsilon = 3$, $D = 0.05$ (blue curve), $\Delta\epsilon = 2$, $D = 50$ (orange curve), and $\Delta\epsilon = 2$, $D = 0.05$ (cyan curve). $\Delta\epsilon_C$ is fixed to 1. The unbalance reaches a maximum for a finite value of $\Delta T = \Delta T^*$, and then goes back to 1 asymptotically for infinite gradients. The value of the maximum increases with $\Delta\epsilon$. *Inset)* ΔT^* as a function of $\Delta\epsilon$ for $D = 50$ (solid gray curve) and $D = 0.05$ (dashed gray curve), $T(0) = 0.2$ and $\Delta E = 2$. The solid black curve is obtained for $D = 50$ and $\Delta\epsilon_C = 2$. The unbalance between energy barriers favors the selection, decreasing the value of ΔT^*. The diffusion enhances the value of the maximum, while increasing the mixing, hence increasing also the value of the optimal gradient at which it is obtained. Moreover, increasing $\Delta\epsilon_C$ the system has lower barriers, while maintaining the same unbalance, so that the energy required to have the maximal selection decreases.

As discussed in the main text, the ratio between the populations of C and B starts from 1 for $\Delta T = 0$ (equilibrium conditions), increases up to a maximum value, and then goes back to 1 asymptotically for infinite gradients.

"Optimal" gradient

The ratio $R_{CB} = P_C/P_B$ reaches its maximum value, *i.e.* the out-of-equilibrium selection is maximized, for a given value of the gradient, ΔT^*, that we name "optimal".

In Fig. S1 we study numerically how the optimal gradient varies as a function of the difference between the two energy barriers $\Delta\epsilon = \Delta\epsilon_B - \Delta\epsilon_C$, which is responsible for the kinetic symmetry-breaking of the system. As $\Delta\epsilon$ increases, ΔT^* decreases, since the symmetry-breaking is enhanced, hence favoring the selection. However, if $\Delta T > \Delta T^*$ the system starts becoming more and more insensitive to the energy barriers, relaxing into a state with all equal populations in the limit of infinite gradient.

We show two curves of $\Delta T^*(\Delta\epsilon)$, for two different values of the diffusion coefficient D. We note that, for a given value of the available energy, ΔT, a stronger diffusion increases the value of ΔT^*, while increasing the maximal selection as well (see main text).

Moreover, decreasing both the barriers, while maintaining the same kinetic symmetry-breaking $\Delta\epsilon$, decreases the energy required to have the maximal selection, i.e. the value of ΔT^*.

Selection under time-periodic variation of temperature

Here, we present the detailed derivation of Eqs. (21), (22) and (23) of the main text. Let us consider the three-state system investigated so far, in contact with a thermal bath whose temperature is periodically varied with time. The transition rates satisfy the following relations:

$$\begin{aligned} k_{A\to B}(t) &= e^{(E_A-E_B)/k_\mathrm{B}T(t)}k_{B\to A}(t) \\ k_{A\to C}(t) &= e^{(E_A-E_C)/k_\mathrm{B}T(t)}k_{C\to A}(t) \\ k_{C\to A}(t) &= e^{\Delta\epsilon/k_\mathrm{B}T(t)}k_{B\to A}(t) \end{aligned} \tag{S25}$$

The temperature is changed according to the following protocol:

$$T(t) = T_\mathrm{m} + \eta_\tau(t)\Delta T \tag{S26}$$

where $\eta_\tau(t)$ is a periodic function of period τ. Moreover, the following constraints hold:

$$\begin{aligned} \frac{1}{\tau}\int_0^\tau \eta_\tau(t)dt &= \overline{\eta_\tau(t)} = 0 \\ \frac{1}{\tau^2}\int_0^\tau\int_0^\tau \eta_\tau(t)\eta_\tau(t')dtdt' &= \overline{\eta_\tau(t)\eta_\tau(t')} = \delta(t-t') \end{aligned} \tag{S27}$$

We can perform two meaningful limits: infinitely fast driving and small gradient expansion.

Infinitely fast driving

This case corresponds to $\tau \to 0$. The probability for each chemical species X will reach a time-periodic state after a transient. Since the driving is infinitely fast, such state will be close to the average value over one period, with the correction scaling with the period:

$$P_X(t) = \overline{P_X} + \tau\Pi_X(t) \tag{S28}$$

Here, $P_X(t)$ is the probability to be in the chemical state X at time t, with $X = A, B, C$. Solving the time-dependent Master Equation order by order, the leading term is:

$$\frac{d\overline{P_X}}{dt} = \sum_Y \left(k_{Y\to X}\overline{P_Y} - k_{X\to Y}\overline{P_X}\right) \tag{S29}$$

Integrating over one period on both sides, we obtain:

$$0 = \sum_Y \left(\overline{k_{Y\to X}}\;\overline{P_Y} - \overline{k_{X\to Y}}\;\overline{P_X}\right) \tag{S30}$$

The time-averaged probability to occupy a given state satisfies, to the leading order in τ, a time-averaged version of detailed balance. Hence, for $\tau \to 0$, the selection is quantified as

$$R_{CB} = \frac{\overline{P_C}}{\overline{P_B}} = \frac{\overline{k_{B\to A}}\;\overline{k_{A\to C}}}{\overline{k_{A\to B}}\;\overline{k_{B\to C}}} \tag{S31}$$

Small gradient expansion

As for the case of a thermal gradient detailed above, we can perform a series expansion in powers of $\delta T = \Delta T/T_\mathrm{m}$ on transition rates and probabilities:

$$\begin{aligned} k_{X\to Y}(t) &= k^{(0)}_{X\to Y} + \delta T k^{(1)}_{X\to Y} + \delta T^2 k^{(2)}_{X\to Y} + \mathcal{O}(\delta T^3) \\ P_X(t) &= P^{(0)}_X(t) + \delta T P^{(1)}_X(t) + \delta T^2 P^{(2)}_X(t) + \mathcal{O}(\delta T^3) \end{aligned} \tag{S32}$$

The normalization condition has to be satisfied independently of the order in δT, so that:

$$\sum_X P_X^{(0)} = 1$$
$$\sum_X P_X^{(n)} = 0 \quad \forall n \tag{S33}$$

Hence, writing the Master Equation up to second order in δT, we have three independent equations:

$$\frac{dP_X^{(0)}}{dt} = \sum_Y \left(k_{Y\to X}^{(0)} P_Y^{(0)} - k_{X\to Y}^{(0)} P_X^{(0)} \right) \tag{S34}$$

$$\frac{dP_X^{(1)}}{dt} = \sum_Y \left(k_{Y\to X}^{(1)} P_Y^{(0)} - k_{X\to Y}^{(1)} P_X^{(0)} + k_{Y\to X}^{(0)} P_Y^{(1)} - k_{X\to Y}^{(0)} P_X^{(1)} \right) \tag{S35}$$

$$\frac{dP_X^{(2)}}{dt} = \sum_Y \left(k_{Y\to X}^{(1)} P_Y^{(1)} - k_{X\to Y}^{(1)} P_X^{(1)} + k_{Y\to X}^{(0)} P_Y^{(2)} - k_{X\to Y}^{(0)} P_X^{(2)} + k_{Y\to X}^{(2)} P_Y^{(0)} - k_{X\to Y}^{(2)} P_X^{(0)} \right) \tag{S36}$$

which are zeroth, first and second order of the expansion, respectively.

Since the zeroth-order transition rates are the same of a system in contact with a bath at temperature $T_{\rm m}$, from Eq. (S34), the zeroth-order probability distribution is the equilibrium solution at temperature $T_{\rm m}$:

$$P_X^{(0)} = \frac{e^{-E_X/k_{\rm B}T_{\rm m}}}{\sum_X e^{-E_X/k_{\rm B}T_{\rm m}}} \tag{S37}$$

for any energy distribution $\{\Delta E_X\}_X$. In our case, $E_A = 0, E_B = E_C = -\Delta E$.

Averaging over one period both sides of Eq. (S35), and exploiting $\overline{\eta(t)} = 0$, we have:

$$0 = \sum_Y \left(k_{Y\to X}^{(0)} \overline{P_Y^{(1)}} - k_{X\to Y}^{(0)} \overline{P_X^{(1)}} \right) \tag{S38}$$

Employing the normalization condition on $P_X^{(1)}$, we have: $\overline{P_X^{(1)}} = 0$, for $X = A, B, C$. Moreover, it will be useful hereafter to derive $\overline{\eta_\tau P_X^{(1)}}$. Moving to the frequency domain, $t \to \omega$, we have:

$$\omega \tilde{P}_X^{(1)} = \tilde{\eta}_\tau F_X + \sum_Y \left(k_{Y\to X}^{(0)} \tilde{P}_Y^{(1)} - k_{X\to Y}^{(0)} \tilde{P}_X^{(1)} \right) \tag{S39}$$

where the tilde represents the Fourier transform. In the fast driving regime, $\omega \gg k^{(0)}$, we obtain:

$$P_X^{(1)} \approx F_X \int dt\, \eta_\tau(t) \tag{S40}$$

and hence:

$$\overline{\eta_\tau P_X^{(1)}} = \frac{1}{\tau}\int_0^\tau dt\, \eta_\tau(t) P_X^{(1)}(t) \approx \frac{F_X}{2}\frac{1}{\tau}\int_0^\tau dt\, \eta_\tau(t) \int_0^t d\theta\, \eta_\tau(\theta) = 0 \tag{S41}$$

Since this term appears by averaging over one period Eq. (S36), when the driving is faster than all chemical transition rates, the first order term does not contribute to the solution up to the second order in δT. Indeed,

$$0 = \sum_Y \left(k_{Y\to X}^{(0)} \overline{P_Y^{(2)}} - k_{X\to Y}^{(0)} \overline{P_X^{(2)}} + \overline{k_{Y\to X}^{(2)}} P_Y^{(0)} - \overline{k_{X\to Y}^{(2)}} P_X^{(0)} \right) \tag{S42}$$

Solving and computing the selection R_{CB} up to the second order leads to the equation shown in the main text:

$$R_{CB} = 1 + \frac{\Delta E \Delta\epsilon}{T_{\rm m}^4}\Delta T^2 \tag{S43}$$

Notice that this equation is obtained in the fast driving/small gradient limit, while a similar one has been derived in the fast diffusion/small gradient approximation. This observation endorses the comparison between driving and diffusion discussed in the main text.

II. SUPPLEMENTARY DISCUSSION: RELATION BETWEEN SELECTION AND DISSIPATION

This section is dedicated to a more in-depth numerical study of the correlation between the unbalance of the populations of species C and B, R_{CB}, and the steady state entropy production, $\dot{S}/\Delta E$ [6, 7], for the two-box model presented in the manuscript.

In Fig. S2 of main text we showed the correlation between the unbalance of the population of C with respect to the one of B, quantified through their ratio R_{CB}, and the entropy production at stationarity, fixing a specific value for each parameter, most notably for the energy barrier $\Delta\epsilon$. Moreover, in the main text we presented only the case of infinite diffusion. Here we discuss the robustness of our results for other choices of the parameters.

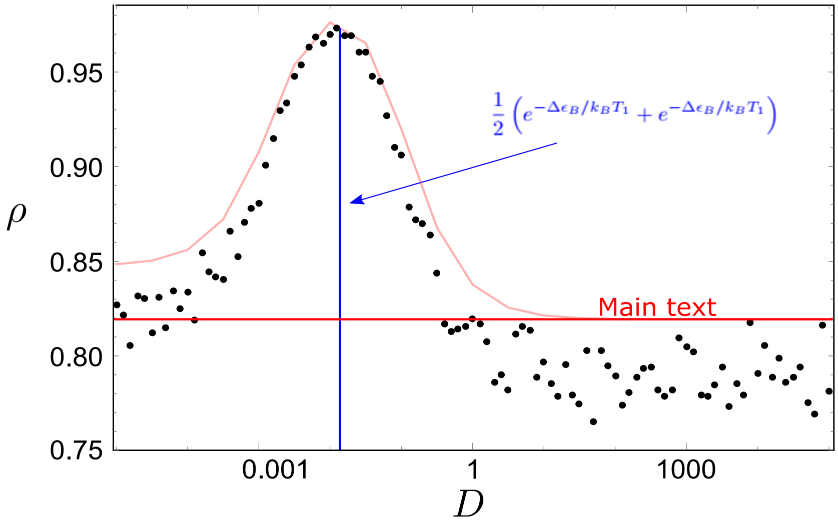

FIG. S2: Correlation coefficient ρ between R_{CB} and $\dot{S}/DE$ as a function of the diffusion coefficient D (the x-axis is in log-scale). The parameters have been set as in the main text of manuscript: $T_{\rm m} = 0.7$, $\Delta\epsilon = 2$ and 10^3 values of ΔE and ΔT, drawn from a Gaussian distribution with mean 1 and 0.2 and standard deviation 0.1 and 0.02, respectively. Each black point indicates the correlation for one realization of ΔE and ΔT with a given and finite D. The shaded red curve indicates ρ for the particular realization shown in Fig. S2 of the main text for different values of the diffusion. The solid horizontal red line is the value reported in the main text for infinite diffusion. The vertical blue line is a graphical comparison between the value at which ρ is maximized and the slowest chemical rate averaged over both boxes.

In Fig. S2, we show that the correlation is not reduced in the presence of finite diffusion, while presenting a maximum for a finite value of D. This observation evidences that we can study only the limit $D \to +\infty$ to obtain useful insights on the system. In particular, it is worth noting that the case presented in the main text is particularly good in terms of correlation, although qualitatively comparable to all the others. As an additional numerical evidence, we observe that the value of diffusion rate D^* at which ρ is maximized is compatible with the slowest chemical rate averaged over both boxes:

$$D^* \approx \frac{1}{2}\left(e^{-\Delta\epsilon_B/k_{\rm B}T_1} + e^{-\Delta\epsilon_B/k_{\rm B}T_1}\right) \tag{S44}$$

indicating an interplay between reaction and diffusion processes. Here, $T_{1,2} = T_{\rm m} \pm \mu_{\Delta T}/2$, with $\mu_{\Delta T}$ indicates the average of the Gaussian distribution from which all the gradients are extracted.

In the main text, we studied the correlation between R_{CB} and $\dot{S}/\Delta E$ fixing the value of $\Delta\epsilon$, $\Delta\epsilon_C$ and $T_{\rm m}$. Moreover, we extracted the value of the gradient from a Gaussian distribution with a given mean $\mu_{\Delta T}$. Here, we study the effect of changing the energy barriers and the average temperature on such a correlation. As a first observation, we know the existence of a maximum for R_{CB} at a critical value ΔT^*, which depends on $T_{\rm m}$, $\Delta\epsilon_B$ and $\Delta\epsilon_C$ (see main text). For small gradients, the correlation is analytically obtained in the main text. For $\Delta T \gg \Delta T^*$ the selection goes to zero since the warm box starts dominating over the cold one. We also discussed that this is a peculiar effect of the discrete two-box model. Hence, we move in a region in which ΔT is close to its optimal value. In Fig. S3 we show the correlation between selection and dissipation for two different choices of $T_{\rm m}$, $\Delta\epsilon_C$ and $\Delta\epsilon$.

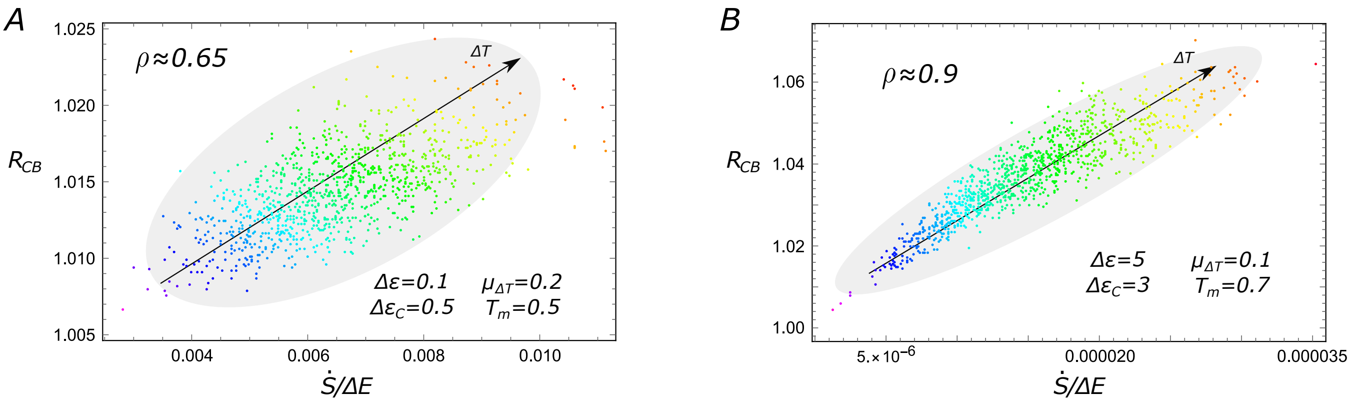

FIG. S3: Correlation between $R_{CB} = P_C/P_B$ and $\dot{S}/\Delta E$ for infinite diffusion, a set of 10^3 values of ΔE drawn from a Gaussian distribution with mean 1 and standard deviation 0.1, a set of 10^3 values of ΔT Gaussian distributed with mean $\mu_{\Delta T}$ and standard deviation 0.02, for different choices of energy barriers $\Delta\epsilon_B$ and $\Delta\epsilon_C$, and average temperature $T_{\rm m}$. ρ is the correlation coefficient. A) $\Delta\epsilon_B = 0.6$, $\Delta\epsilon_C = 0.5$, $\mu_{\Delta T} = 0.2$ and $T_{\rm m} = 0.5$. The points exhibit a good positive correlation, despite the ratio $\Delta T/T_{\rm m} \approx 0.4$. B) $\Delta\epsilon = 1$ and $\mu(\Delta T) = 1$, exhibiting ρ close to 1. The Inset present the correlation in the particular case $\Delta T = 1.2$. C) $\Delta\epsilon_B = 8$, $\Delta\epsilon_C = 3$, $\mu(\Delta T) = 0.1$ and $T_{\rm m} = 0.7$. Here the correlation is stronger than the one for Panel A, according to the fact that we are closer to the small gradient regime.

III. SUPPLEMENTARY NOTE 1: SELECTION IN A BRANCHING TREE OF REACTIONS

In the main text we inspected how global localization phenomena may arise in a chain of connected reactions in presence of fast diffusion. Another interesting and quite simple example in which the kinetic symmetry-breaking plays a leading role is provided by a branching tree of chemical reactions from lower to higher energies, as sketched in Fig. S4A. Also in this case we consider the infinitely fast diffusion limit. When no temperature gradient is applied, the population of each state follows the Boltzmann distribution, and progressively deviates from it as the temperature gradient is increased, the role of the reaction rates becoming progressively more important. For $\Delta T \to 2T_{\rm m}$, the warm box becomes dominant, and the population tends to an equilibrium distribution with temperature $T_{\rm m} + \Delta T/2$. As an indicator of this feature, we compute the Kendall correlation coefficient [8] between $R_{C_k B}$ and E_k for each state. As expected, it is equal to -1 at equilibrium, whereas it lowers when increasing ΔT, and eventually it goes back to -1 for large values of the gradient (see Fig. S4B).

Another possible quantification of how much the system is deviating from equilibrium is provided by the variance of the ratio between populations of iso-energetic states, V_P, defined as

$$V_p = \mathrm{var}_{k,k'}\left(\frac{P_{C_k}}{P_{C_{k'}}}\delta_{E_k,E_{k'}}\right) \tag{S45}$$

where the subscripts k and k' indicates that the variance is performed over the whole possible pair of states. As for the Kendall correlation coefficient, this quantity is 0 when the system follows an equilibrium distribution, since the population depends only on the energy, while it increases for intermediate values of ΔT (see Fig. S4B).

A side remark is that, as expected, the population of all states is shifted towards higher values of E_k when the available thermal energy is increased (see Fig. S4B).

Again, the velocity of each reaction with respect to its adjacent ones is not sufficient to determine the stationary population of the states involved in it. In fact, the most important ingredient determining which states $\{C_k^*\}$ will have a net increase in their populations, in the non-equilibrium steady state, is once again the weight of the path connecting each C_k^* to a reference state (B in this case; clearly, as it can be shown, the ranking of the states does not depend on the chosen reference). Stated otherwise, the system select the fastest paths from lower (B) to higher energies (C_k), ideally providing a natural identification of the best possible tree of reactions which lead to the most stable metastable states.

IV. SUPPLEMENTARY NOTE 2: THERMOPHORESIS IN A TWO-SPECIES TWO-BOX MODEL

In the main text, we discussed the emergence of thermophoresis [9, 10], *i.e.* the accumulation of particles to the hot or cold side of a gradient, as an inevitable consequence of the law of thermodynamics. In particular, we have

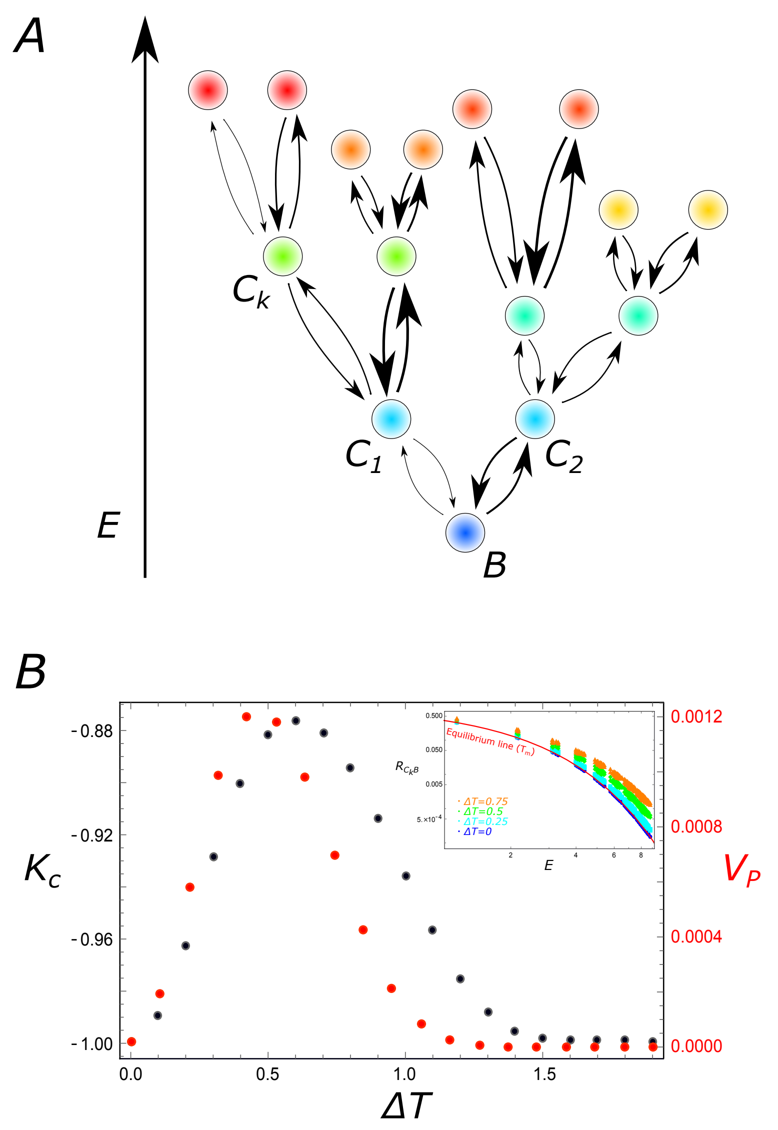

FIG. S4: A) A chain of reactions branching from lower to higher energies with a branching ratio equal to 2. Same colors correspond to the same energy, and the thicker is the arrow, the faster the reaction. For the sake of simplicity, we set the energy of each offspring to be the same. B) Kendall correlaion coefficient K_c between $R_{C_k B}$ and E_k as a function of ΔT in black. As the system is driven away from equilibrium the kinetics starts being important to determine the steady distribution. For large values of the gradient the warm box dominates, and the system reach the equilibrium distribution associated with temperature $T_{\mathrm{m}} + \Delta T/2$. Analogously, an optimal value for the gradient is found studying the variance of the ratio between populations of iso-energetic states, V_p, defined in Eq. (S45) (red dots). *Inset* - The shift of the distribution towards higher energies as a function of ΔT is quantified through the quantity $R_{C_k B}$, which is the ratio between the population of the state C_k and the ground (reference) state B.

shown that this phenomenon may appear in a three-state two-box model, where the fastest state (C) is selected in non-equilibrium steady state, introducing an unbalance between the transport coefficients of each species. We also provided an interpretation of thermophoresis as a selection process *in real space*, rather than in the state of the species.

Here, we want to point out that thermphoresis is independent of the selection of the fastest species, being intimately related to the kinetic-symmetry breaking of the transport properties instead. To this aim, we consider a two species two-box model, as shown in Fig. S5A. Here, by construction, the selection of states is prevented: there is trivially only one dissipative cycle in the system passing through all the states.

However, if $d_A \neq d_B$, an unbalance between $P_1 = P(A_1)+P(B_1)$ and $P_2 = P(A_2)+P(B_2)$ is obtained (see Fig. S5B), which is the emerging behaviour reminiscent of thermophoresis we are looking for. Indeed, to this unbalance we can

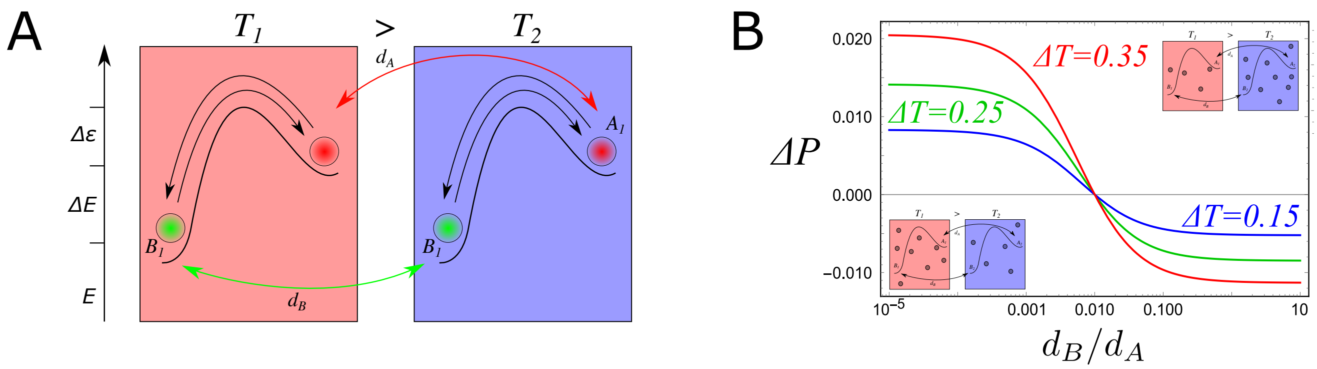

FIG. S5: A) A two-state chemical system diffusing in a temperature gradient, modeled as two connected boxes at different temperatures, $T_1 > T_2$. The transport coefficient of each species is equal to d_X, where $X = A, B$. B) Unbalance of populations $\Delta P = P_2 - P_1$ as a function of $\alpha = d_B/d_A$ (in log-scale) for three different values of the gradient. The system can accumulate both on the warm or cold side depending on the ratio between the transport rates. The higher is the available energy in the form of a thermal gradient, the greater will be the unbalance. The parameters have been set as follows: $T = 0.7, \Delta E = 0.1, d_A = 0.01$, and $k_\mathrm{B} = 1$ for sake of simplicity.

associate a Soret coefficient [11–13], following the same strategy explained in the main text. In the limit of small diffusion, we have the following simple expression as a function of energy difference ΔE, temperature T_2 and ratio of transport rates d_B/d_A:

$$S_\mathrm{T} = \frac{(1 - d_B/d_A)e^{-\Delta E/k_\mathrm{B}T_2}}{(1 + e^{-\Delta E/k_\mathrm{B}T_2})((d_B/d_A)e^{-\Delta E/k_\mathrm{B}T_2} + 1)k_\mathrm{B}T_2^2} \tag{S46}$$

[1] C. Gardiner, Stochastic methods, vol. 4 (Springer Berlin, 2009).
[2] S. Bo and A. Celani, Physics reports **670**, 1 (2017).
[3] M. Vucelja, O. Raz, O. Hirschberg, and I. Klich, arXiv preprint arXiv:1711.05829 (2017).
[4] D. Mandal and C. Jarzynski, Journal of Statistical Mechanics: Theory and Experiment **2011**, P10006 (2011).
[5] D. M. Busiello, J. Hidalgo, and A. Maritan, arXiv preprint arXiv:1810.01833 (2018).
[6] J. Schnakenberg, Reviews of Modern physics **48**, 571 (1976).
[7] D. M. Busiello, J. Hidalgo, and A. Maritan, Physical Review E **96**, 062110 (2017).
[8] M. G. Kendall (1948).
[9] S. Duhr and D. Braun, Proceedings of the National Academy of Sciences **103**, 19678 (2006).
[10] R. Piazza and A. Parola, Journal of Physics: Condensed Matter **20**, 153102 (2008).
[11] J. K. Platten, Journal of applied mechanics **73**, 5 (2006).
[12] W. Koehler and K. I. Morozov, Journal of Non-Equilibrium Thermodynamics **41**, 151 (2016).
[13] M. Rahman and M. Saghir, International Journal of Heat and Mass Transfer **73**, 693 (2014).
